# Decrease of breast cancer cell invasiveness by sodium phenylacetate (NaPa) is associated with an increased expression of adhesive molecules

**DOI:** 10.1054/bjoc.2000.1648

**Published:** 2001-03

**Authors:** M Vasse, D Thibout, J Paysant, E Legrand, C Soria, M Crépin

**Affiliations:** Laboratoire DIFEMA, Groupe de Recherche MERCI, Faculté de Médecine et Pharmacie de Rouen, 22 Bd Gambetta, Rouen Cedex, 76183, France; équipe d'Oncologie Cellulaire et Moléculaire, SMBH Université de Paris 13, UPRES 2360, 74 rue Marcel Cachin, Bobigny Cedex, 93017, France; U353, Hôpital St Louis, 1 avenue Claude Vellefaux, Paris, Cedex 10, 75475, France

**Keywords:** sodium phenylacetate, invasiveness, breast cancer, adhesive molecules

## Abstract

Sodium phenylacetate (NaPa), a non-toxic phenylalanine metabolite, has been shown to induce in vivo and in vitro cytostatic and antiproliferative effects on various cell types. In this work, we analysed the effect of NaPa on the invasiveness of breast cancer cell (MDA-MB-231, MCF-7 and MCF-7 ras). Using the highly invasive breast cancer cell line MDA-MB-231, we demonstrated that an 18-hour incubation with NaPa strongly inhibits the cell invasiveness through Matrigel (86% inhibition at 20 mM of NaPa). As cell invasiveness is greatly influenced by the expression of urokinase (u-PA) and its cell surface receptor (u-PAR) as well as the secretion of matrix metalloproteinases (MMP), we tested the effect of NaPa on these parameters. An 18-hour incubation with NaPa did not modify u-PA expression, either on MDA-MB-231 or on MCF-7 and MCF-7 ras cell lines, and induced a small u-PA decrease after 3 days of treatment of MDA-MB-321 with NaPa. In contrast, an 18 h incubation of MDA-MB-231 increased the expression of u-PAR and the secretion of MMP-9. As u-PAR is a ligand for vitronectin, a composant of the extracellular matrix, these data could explain the increased adhesion of MDA-MB-231 to vitronectin, while cell adhesivity of MCF-7 and MCF-7 ras was unmodified by NaPa treatment. NaPa induced also an increased expression of both Lymphocyte Function-Associated-1 (LFA-1) and Intercellular Adhesion Molecule-1 (ICAM-1), which was obvious from 18 hour incubation with NaPa for the MDA-MB-231 cells, but was delayed (3 days) for MCF-7 and MCF-7 ras. Only neutralizing antibodies against LFA-1 reversed the decreased invasiveness of NaPa-treated cells. Therefore we can conclude that the strong inhibition of MDA-MB-231 invasiveness is not due to a decrease in proteases involved in cell migration (u-PA and MMP) but could be related both to the modification of cell structure and an increased expression of adhesion molecules such as u-PAR and LFA-1. © 2001 Cancer Research Campaign http://www.bjcancer.com


*British Journal of Cancer* (2001) **84**(6), 802–807
© 2001 Cancer Research Campaign
doi: 10.1054/ bjoc.2000.1648, available online at http://www.idealibrary.com on IDEAL®

http://www.bjcancer.com

### Decrease of breast cancer cell invasiveness by sodium phenylacetate (NaPa) is associated with an increased expression of adhesive molecules

**M Vasse[1], D Thibout[2], J Paysant[1], E Legrand[1], C Soria[1,3] and M Crépin[2]**

[1]Laboratoire DIFEMA, Groupe de Recherche MERCI, Faculté de Médecine et Pharmacie de Rouen, 22 Bd Gambetta, 76183 Rouen Cedex, France; [2]UPRES 2360, équipe d'Oncologie Cellulaire et Moléculaire, SMBH Université de Paris 13, 74 rue Marcel Cachin, 93017 Bobigny Cedex, France; [3]U353, Hôpital St Louis, 1 avenue Claude Vellefaux, 75475 Paris Cedex 10, France

**Summary** Sodium phenylacetate (NaPa), a non-toxic phenylalanine metabolite, has been shown to induce in vivo and in vitro cytostatic and antiproliferative effects on various cell types. In this work, we analysed the effect of NaPa on the invasiveness of breast cancer cell (MDA-MB-231, MCF-7 and MCF-7 ras). Using the highly invasive breast cancer cell line MDA-MB-231, we demonstrated that an 18-hour incubation with NaPa strongly inhibits the cell invasiveness through Matrigel (86% inhibition at 20 mM of NaPa). As cell invasiveness is greatly influenced by the expression of urokinase (u-PA) and its cell surface receptor (u-PAR) as well as the secretion of matrix metalloproteinases (MMP), we tested the effect of NaPa on these parameters. An 18-hour incubation with NaPa did not modify u-PA expression, either on MDA-MB-231 or on MCF-7 and MCF-7 ras cell lines, and induced a small u-PA decrease after 3 days of treatment of MDA-MB-321 with NaPa. In contrast, an 18 h incubation of MDA-MB-231 increased the expression of u-PAR and the secretion of MMP-9. As u-PAR is a ligand for vitronectin, a composant of the extracellular matrix, these data could explain the increased adhesion of MDA-MB-231 to vitronectin, while cell adhesivity of MCF-7 and MCF-7 ras was unmodified by NaPa treatment. NaPa induced also an increased expression of both Lymphocyte Function-Associated-1 (LFA-1) and Intercellular Adhesion Molecule-1 (ICAM-1), which was obvious from 18 hour incubation with NaPa for the MDA-MB-231 cells, but was delayed (3 days) for MCF-7 and MCF-7 ras. Only neutralizing antibodies against LFA-1 reversed the decreased invasiveness of NaPa-treated cells. Therefore we can conclude that the strong inhibition of MDA-MB-231 invasiveness is not due to a decrease in proteases involved in cell migration (u-PA and MMP) but could be related both to the modification of cell structure and an increased expression of adhesion molecules such as u-PAR and LFA-1. © 2001 Cancer Research Campaign http://www.bjcancer.com

**Keywords**: sodium phenylacetate; invasiveness; breast cancer; adhesive molecules

Isoprenoid synthetic pathway inhibitors and farnesyl transferase inhibitors represent a new class of anticancer drugs, very promising to block the tumour growth without important toxicity. Among these molecules sodium phenylacetate (NaPa), a product of phenylalanine metabolism, has been shown to induce cytostasis (Prasanna et al, 1995) and differentiation in a variety of tumour models (Samid et al, 1992a, 1992b). Thus, it was previously shown by us and others that NaPa treatment can induce tumour cell apoptosis alone (Adam et al, 1995) or in association with tamoxifen (Adam et al, 1997). These antiproliferative molecules induce DNA fragmentation, cytochrome C release and caspase 3 activation preferentially in ras transformed cells (Suzuki et al, 1998). In vivo studies on animal models demonstrated an antitumoral effect of NaPa and derivatives. Thus, MCF-7 ras tumour development in nude mice was blocked by NaPa treatment (Adam et al, 1995). This NaPa antitumoral activity can be related not only to antiproliferative effect on MCF-7 ras but also to stromal paracrine effects. In this context, we have shown that NaPa treatment modulated the synthesis and secretion of autocrine and paracrine growth factors secreted by MCF-7 ras cells (Thibout et al, 1998). In our in vivo previous study, we have observed that NaPa induced, in mammary MCF-7 ras tumours, a stroma development, a tumour angiogenesis reduction and a decrease of cell invasiveness in adjacent tissues (Adam et al, 1997). These observations led us to investigate the u-PA/u-PAR protease system clearly involved in the pericellular proteolysis necessary to tumour cell migration (Holst-Hansen et al, 1996). Tumour cell adhesion to extracellular-matrix (ECM) proteins was also shown to be important for the tumour invasive process (Tatsumi et al, 1996). Among these proteins, we focused our attention on fibronectin, a major protein found in almost all the tissues, and vitronectin which uses u-PAR as a receptor (Carriero et al, 1997). In order to become more invasive, tumour cells must stabilize adhesive interactions that prevent detachment at secondary sites. Intercellular adhesion molecules like ICAM-1, LFA-1 and MAC-1 (CD11b) were shown to be associated with phenotypes of various tumour cell types (Hayashi et al, 1997). For these reasons, we investigated these 3 adhesion molecules in 3 cell lines, representative of 3 different phenotypes (MCF-7, MCF-7 ras and MDA-MB-231) treated with increasing concentrations of NaPa.

*Received 7 June 2000*
*Revised 23 November 2000*
*Accepted 29 November 2000*

*Correspondence to:* M Crépin

### MATERIALS AND METHODS

### Antibodies and reagents

The monoclonal antibody (mAb) to u-PA which recognizes the α-chain of u-PA (#3639) was from American Diagnostica (Greenwich, USA). Fluorescein isothiocyanate-labelled

(FITC-labelled) antibodies to MAC-1 (CD11b), LFA-1 (CD11a) and F(ab′)2 fraction of goat anti-mouse IgG1 were from Immunotech (Marseille, France). The FITC-labelled mAb to ICAM-1 (CD104) was from R&D Systems (Abington, UK) and the FITC-labelled F(ab′)2 fraction of swine anti-rabbit were purchased from Dako (Trappes, France). Non-labelled mAb antibodies to CD104 (R&D Systems) and LFA-1 (Immunotech) were used for neutralizing assays. The polyclonal antibody to PAI-1 was kindly provided by R Lijnen (Leuven, Belgium). Vitronectin was from Serbio laboratories (Gennevilliers, France), fibronectin was from Sigma (St Louis, USA) and NaPa from SERATEC (France).

### Cell cultures

MDA-MB-231 were cultured in RPMI 1640 (Eurobio, les Ulis, France) supplemented with 10% fetal calf serum (FCS), 1 mM L-glutamine, 100 IU $ml^{-1}$ penicillin, 100 μg $ml^{-1}$ streptomycin (Gibco Brl, NY, USA) MCF-7 and MCF-7 ras were cultured in DMEM supplemented with FCS (10%), L-glutamine (2 mM), and antibiotics as above.

### Cell invasiveness through Matrigel

8-μm-diameter pore Transwell (Dutcher, Brumath, France) were coated with 500 μl of Matrigel (Beckton-Dickinson Europe, Meylan, France) diluted at 100 μg $ml^{-1}$. Breast tumour cells were detached with trypsin (0.05%, w/v) (Sigma), washed twice with phosphate buffer saline (PBS) (Eurobio) and $2 \times 10^5$ cells in RPMI 1640 with 0.2 mg $ml^{-1}$ bovine serum albumin (BSA) (Sigma) were seeded in the upper chamber of the Matrigel-coated insert. The lower chamber was filled with 1 ml of RPMI 1640 together with 2 mg $ml^{-1}$ BSA and 20 ng $ml^{-1}$ basic fibrobast growth factor (bFGF) (R&D Systems). After 18 hours of incubation, the non-migrated cells in the upper chamber were gently scraped, and the adherent cells present on the lower surface of the insert were coloured by May-Grünewald-Giemsa and counted by light microscopy, 10 fields (magnification ×200) were counted for each insert. For neutralizing assay, the cells were incubated for 30 min at 4˚C with 100 μg of the neutralizing mAb before being seeded in the upper chamber of the insert. Controls were incubated with an irrelevant IgG1 antibody (Immunotech) at the same concentration.

### Flow cytometry analysis

Flow cytometry analysis were performed on non-permeabilized cells, as previously described (Paysant et al, 1998). ICAM-1 (CD104), LFA-1 (CD11a) and MAC-1 (CD11b) expression on breast cancer cells were determined by direct immunofluorescence, while u-PA and PAI-1 were detected by indirect immunofluorescence. Tumour cells were detached by a non-enzymatic cell dissociation solution (Sigma) and the cells were washed twice in cold PBS. Approximately $5 \times 10^5$ cells were incubated for 15 minutes at 4˚C with 10 μl of the specific antibodies (1 mg $ml^{-1}$). After 2 washes, the cell suspension was immediately analysed in a flow cytometer (EPICS XL-MCL, Coulter, USA) when antibody were directly conjugated to FITC, while another 15 minutes incubation with a FITC anti-mouse IgG1 antibody or an anti-rabbit antibody (10 μg $ml^{-1}$) was carried out respectively for the detection of u-PA and PAI-1 antibody before cytometry analysis. Data are expressed either as the percentage of fluorescent cells or as the specific mean channel fluorescence intensity (MFI). Specific MFI was calculated for each sample by subtracting the background MFI produced by an irrelevant antibody from the MFI value generated by the specific antibody.

### Measurement of u-PAR antigen

The adherent cells were washed twice with PBS and lysed in PBS containing 0.1% Triton X-100 and sonicated (Vibracell Bioblock, Illkirch, France) at 60 Hz for 20 seconds. u-PAR antigen in the lysates was measured by an ELISA assay (Imubind kit u-PAR, American Diagnostica), according to the manufacturer's instructions. The proteins in the cell lysates were assayed by the method of Bradford (1976). Results were expressed in ng of u-PAR $mg^{-1}$ of proteins.

### SDS-polyacrylamide gel electrophoresis zymography

Electrophoresis was performed either on 5 μl of conditioned medium or on 7 μg proteins of the lysate, in 7.5% polyacrylamide gels containing 10% sodium dodecyl sulphate (SDS) and gelatin (1 mg $ml^{-1}$) under non-reducing conditions. After electrophoresis, SDS was removed from gels by washing for 1 hour in 2.5% Triton X-100 at room temperature. Gelatinase activity was revealed overnight in a buffer containing 50 mM Tris-HCl, 5 mM $CaCl_2$, pH 7.6. The gels were stained with Coomassie blue R250 (0.25%) and gelatinolytic activity was evidenced as clear bands against the blue background of stained gelatin.

### Adhesion assay

Adhesion assay was performed as previously described (Paysant et al, 1998), with minor modifications. Tissue culture plates (96 wells) were prepared as follows: 50 μl per well of vitronectin (0.5 μg $ml^{-1}$) or fibronectin (100 μg $ml^{-1}$) were incubated overnight at 4˚C. Following incubation, plates were washed in PBS and non-specific binding sites were blocked with 1% BSA in PBS for 2 hours at 37˚C. Plates were then washed twice in PBS prior to the plating of the cells. Tumour cells were detached by trypsin (0.05%, w/v), washed twice in PBS and 100 μl of a $2\times10^6$ $ml^{-1}$ cell suspension were placed per well in triplicate in vitronectin or fibronectin coated wells. After a 2 hour incubation, non-adherent cells were removed by 3 rinses in PBS, and adherent cells were quantified by their acid phosphatase activity on paranitrophenyl phosphate (1 mg $ml^{-1}$) in sodium acetate buffer pH 5.5. After 6 h the reaction was stopped with NaOH 1N and measurement of absorbance at 405 nm was performed in an automated plate reader (Dynatech, Saint Quentin, France).

### Statistical analysis

Statistical significance was determined by the ANOVA test using the InStat Software (Sigma).

### RESULTS

### Effect of NaPa on the invasiveness of MCF-7, MCF-7 ras and MDA-MB-231 cells

In order to investigate the effect of NaPa on the tumour invasive processes, we have compared on one hand the invasiveness of 3

© 2001 Cancer Research Campaign

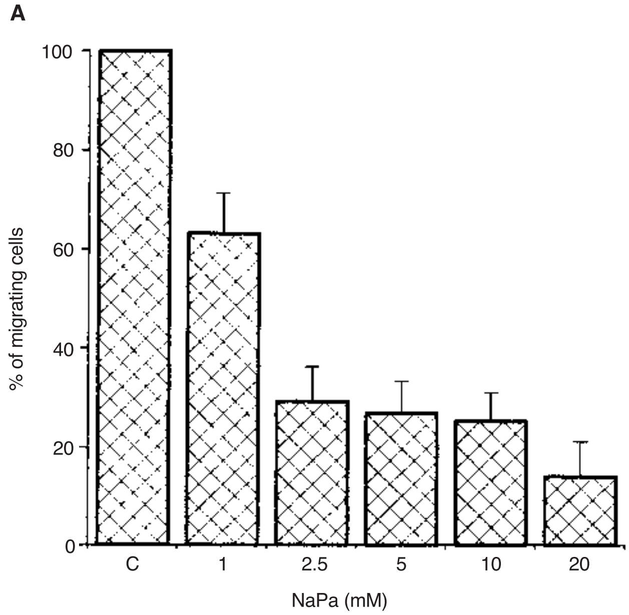

**Figure 1** Effect of increasing concentrations of NaPa on MDA-MB-231 invasiveness through Matrigel. MDA-MB-231 were treated for 18 hours by increasing concentrations of NaPa. After trypsination and 2 washes in PBS, $2 \times 10^5$ cells were added in Transwell coated with Matrigel and bFGF (20 ng/ml) was present in the lower part of the invasion chamber (see Materials and Methods section). After an 18 hour incubation at 37°C, the cells in the upper part of the invasion chamber were gently detached, and cells which had traversed the filter (pore 8 µ) were counted by light microscopy after May-Grünewald coloration. 10 fields (magnification ×200) were counted for each insert.

**Table 1** Expression of uPA and cell adhesion molecules (ICAM-1, LFA-1, MAC-1) on MDA-MB-231, MCF-7 and MCF-7 ras cells

| | % of positive cells | | |
|---|---|---|---|
| | MDA-MB-231 | MCF-7 | MCF-7ras |
| uPa | 94.8±4.1 | 14.4±3.8 | 24.3±5.3 |
| ICAM-1 | 78.8±4.5 | 15.6±5.5 | 36±4.8 |
| LFA-1 | 40±5.2 | 28.3±5 | 9.6±3.8 |
| MAC-1 | 11±1.1 | 5.9±1.5 | 4.4±1.2 |

These different molecules were detected by flow cytometry. For each antigen analysed the percentage of positive cells was recorded.

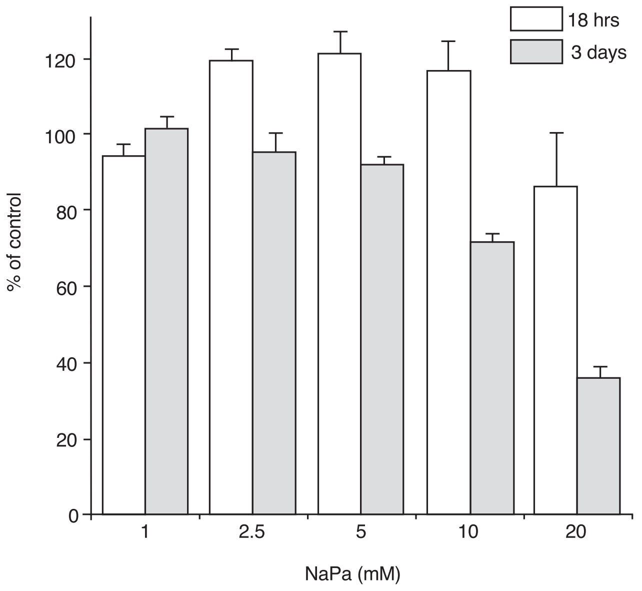

**Figure 2** Effect of increasing concentrations of NaPa on the expression of uPA at the surface of MDA-MB-231. MDA-MB-231 were incubated for 18 hours or 3 days with increasing concentrations of NaPa, and uPA expressed at the membrane was analysed by flow cytometry. The Mean Fluorescence Intensity (MFI) represents the mean intensity of labelling of the positive cells. u-PA was detected by indirect immunofluorescence using a FITC-conjugated antimouse antibody. Data are expressed as the percentage (as compared to the controls) of the MFI ± SEM of 3 separate experiments cell lines with different phenotypes and on the other hand the u-PA and u-PAR system involved in the invasivity of mammary tumour cells. Under our conditions, MCF-7 and MCF-7 ras were weakly invasive in Matrigel test. This is in agreement with other reports (Holst-Hansen et al, 1996; Hazan et al, 2000). In contrast, under the same conditions MDA-MB-231 cells were highly invasive. In 8 µ Transwell coated with Matrigel, 7% of MDA-MB-231 cells migrated after 18 h of incubation. In the presence of increasing concentrations of NaPa, this migration was dose dependently inhibited (Figure 1). With 2.5 mM NaPa the percentage of migratory cells was reduced to 29.5% and for higher NaPa concentrations, NaPa inhibited almost totally the invasion (86% inhibition for 20 mM). Consequently, we investigated u-PA and its receptor (u-PAR) as well as its inhibitor, Plasminogen Activator Inhibitor-1 (PAI-1) which are clearly responsible for tumoral cell invasion.

### Effect of NaPa on u-PA expression in MCF-7, MCF-7 ras and MDA-MB-231 cells

The percentage of u-PA positive cells, analysed by flow cytometry, was very different according to the cancer cell types. u-PA was highly expressed in most of MDA-MB-231 cells (94.8% of positive cells) as compared to MCF-7 cells (14.4%) and MCF-7 ras cells (24.3%) (Table 1). Despite the decrease of cell invasiveness, the NaPa treatment of MDA-MB-231, for 18 h, did not modify the expression of u-PA. Only a 3-day treatment with NaPa at 20 mM reduced the u-PA expression, from 94.8% to 48% of positive cells, and the amount of u-PA expressed by positive cells was reduced to 36.2% (Figure 2). This decrease was highly significant ($P < 0.001$). In contrast, the same NaPa treatment, even after 3 days, did not affect the u-PA expression in MCF-7 and MCF-7 ras cells (data not shown). Whereas MCF-7 and MCF-7 ras do not contain significant amount of PAI-1, this inhibitor is synthetized by MDA-MB 231 and more than 50% of MDA-MB-231 cells expressed PAI-1 at their surface. NaPa treatment had no effect neither on PAI-1 secreted in the medium nor on PAI-1 expressed at cell membrane (data not shown).

### Effect of NaPa on MMP-9 secretion by MCF-7, MCF-7 ras and MDA-MB-231 cells

Despite the decrease in cell invasiveness through Matrigel by NaPa treatment, the amount of MMP-9 either in the conditioned medium or in cell lysates of MDA-MB-231 was increased when treated with NaPa (Figure 3). In our working conditions, we were unable to detect significant amount of MMP in the conditioned medium or in the lysate of MCF-7 and MCF-7 ras cells (data not shown).

© 2001 Cancer Research Campaign

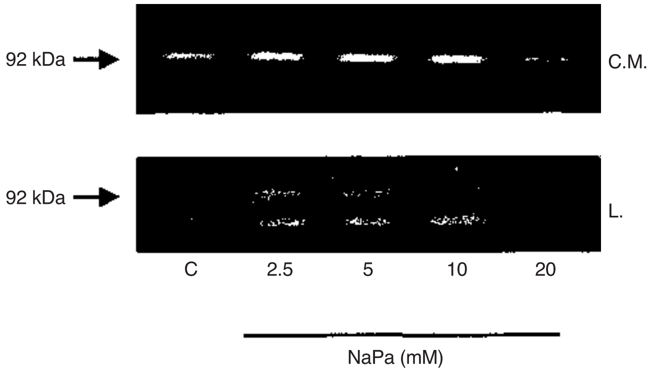

**Figure 3** Analysis of metalloproteinases secreted in conditioned medium or lysates of MDA-MB-231. MDA-MB-231 were incubated in serum-free medium for 48 hours with increasing concentrations of NaPa, and the supernatants or the lysates were analysed by zymography as described in Materials and methods. Molecular weights were determined using pre-stained standards (Bio-Rad). CM: Conditioned Medium; L: lysates; C: concentrations

### Effect of NaPa on the u-PAR content of MCF-7, MCF-7 ras and MDA-MB-231 cells

The total amount of u-PAR was measured by ELISA, using an antibody which recognizes both free and u-PA associated u-PAR. MCF-7 and MCF-7 ras have relatively low amounts of u-PAR (respectively 0.52 ± 0.07 and 0.41 ± 0.04 ng $mg^{-1}$ proteins) as compared to MDA-MB 231 (5.3 ± 1.04 ng $mg^{-1}$ of proteins). A NaPa treatment of 18 h increased dose-dependently the amount of u-PAR in MDA-MB-231 cells whereas it did not change those of MCF-7 and MCF-7 ras cells, even after 3 days (Table 2).

### Effect of NaPa on tumour cell adhesion to extracellular matrix proteins

After 18 h of treatment with 20 mM NaPa, MDA-MB-231 cells take a spindle-shape and detached from the plastic surface. Despite this change of morphology, the viability of the adherent cells was unaltered, as estimated by the blue trypan exclusion test or the absence of increased incorporation of propidium iodide. Lower concentrations did not significantly modify cell shape or adhesivity. In contrast, NaPa treatment did not modify significantly the adhesion and the morphology of MCF-7 and MCF-7 ras cells (data not shown). Since tumour cells express receptors for fibronectin and vitronectin (among them u-PAR) which are extracellular matrix proteins present in tumours (Loridon-Rosa et al, 1988), we compared the adhesivity of the 3 cell lines to these proteins. The cell adhesivities of the 3 cell lines were similar. On vitronectin, an 18 h NaPa treatment increased significantly ($P$ = 0.002) the MDA-MB-231 cell adhesion (Figure 4), whereas it had no effect on the adhesion of MCF-7 and MCF-7 ras cells (data not shown). On fibronectin, NaPa, even at high concentrations, did not modify the adhesion of the 3 cell types (data not shown).

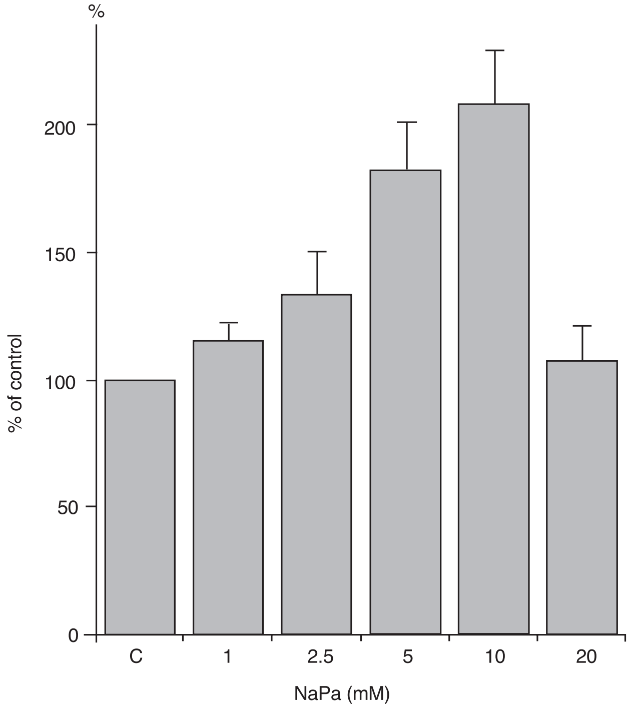

**Figure 4** Effect of increasing concentrations of NaPa on the adhesion of MDA-MB-231, MCF-7 and MCF-7 ras on vitronectin. The tumour cells were incubated for 18 hours in the presence of increasing concentrations of NaPa, then detached by mild trypsination and washed twice with PBS. $2.5 \times 10^5$ cells were incubated for 3 hours in vitronectin coated wells, non-adherent cells were removed by 2 washes in PBS. Adherent cells were quantified by their acid phosphatase activity (see Materials and Methods)

### Effect of NaPa on cell adhesion LFA-1, MAC-1 and ICAM-1 molecules

We compared the 3 cancer cell phenotypes for the expression of these integrins involved in the tumour cell adhesion. The expression of MAC-1 was low in the 3 cell types and only a small percentage of cells were positive for this antigen. As already observed for u-PAR, the expression of MAC-1, LFA-1 and ICAM-1 was higher in MDA-MB 231 cells than in MCF-7 and MCF-7 ras (Table 1). An 18 h

**Table 2** Effect of NaPa on u-PAR synthesis by MDA-MB-231, MCF-7 and MCF-7 ras

| Cell lines | NaPa (mM) | | | | | | |
|---|---|---|---|---|---|---|---|
| | 0 | 1 | 2.5 | 5 | 10 | 20 | ANOVA |
| MDA-MB-231 | 5.3±0.61 | 6±0.56 | 5.5±0.31 | 7.7±0.82 | 8.2±0.61 | 8±0.46 | $P < 0.01$ |
| MCF-7 | 0.52±0.06 | 0.54±0.01 | 0.6±0.23 | 0.81±0.24 | 0.95±0.21 | 0.62±0.05 | NS |
| MCF-7ras | 0.41±0.03 | 0.36±0.05 | 0.44±0.08 | 0.51±0.13 | 0.48±0.11 | 0.4±0.13 | NS |

u-PAR was measured by an ELISA in the lysates of the tumour cells. Cells were treated for 18 hours with NaPa for MDA-MB-231, and for 3 days for MCF-7 and MCF-7 ras. Results are expressed as ng of u-PAR $mg^{-1}$ proteins. (Mean ± SEM of 3 separate experiments). NS = non-significant.

*© 2001 Cancer Research Campaign*

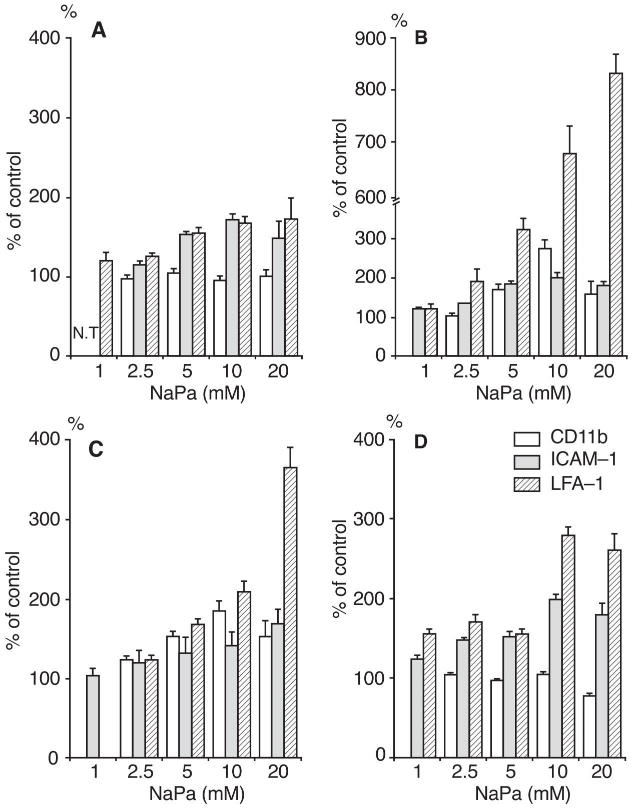

**Figure 5** Effect of increasing concentrations of NaPa on the expression of CD11b(MAC-1) (empty bars), ICAM-1 (grey bars) or LFA-I (hatched bars) of MDA-MB -231 (**A,B**), MCF-7 (**C**), MCF-7ras (**D**). MDA-MB-231 were treated either 18 hours (**A**) or 3 days (**B**) by NaPa, whereas MCF-7 and MCF-7 ras were treated for 3 days. ICAM-1 and MAC-I were detected by flow cytometry by direct immunofluorescence, whereas LFA-1 was detected by indirect immunofluorescence, using a FITC-conjugated anti-mouse antibody. Data are expressed as the percentage ( as compared to the controls) of the MFI ± S.E.M of 3 separate experiments treatment of MDA-MB 231 cells with NaPa increased dose-dependently the expression of both ICAM-1 and LFA-1, whereas MAC-1 was unmodified (Figure 5A). When these cells were treated for 3 days with NaPa, an increase of the 3 adhesion molecules was observed, particularly pronounced for LFA-1. An increase of these 3 adhesion molecules was also observed on MCF-7 and MCF-7 ras, but only after a 3 day treatment with NaPa (Figure 5C and D).

### Consequences of the increase of LFA-1 and ICAM-1 on MDA-MB-231 invasiveness through Matrigel

In order to analyse the involvement of the increased expression of LFA-1 and ICAM-1 in MDA-MB-231 invasion through Matrigel, we performed invasion assay using functional neutralizing antibodies against these molecules. The decreased invasiveness of MDA-MB-231 induced by 20 mM of NaPa was unmodified by the pretreatment with a neutralizing mAb against ICAM-1 while the pretreatment with the neutralizing mAb against LFA-1 partially restores the invasiveness of the NaPa-treated cells (Table 3).

### DISCUSSION

In this paper, we have shown that increasing concentrations of NaPa strongly reduces the invasiveness of the highly invasive MDA-MB-231 cell line. In order to analyse the mechanisms involved in this inhibition, we firstly analysed the expression of u-PAR/u-PA system expressed by these cells and the secretion of MMP-9, since these enzymes are known to facilitate cancer cells invasion (Ossowski, 1992; Bianchi et al, 1994). By comparing MDA-MB-231 to MCF-7 and MCF-7 ras cells we have shown that this cell line expresses high levels of u-PA and secretes MMP-9 in the conditioned medium. In addition only MDA-MB 231 cells secrete and express at their surface PAI-1 which is also involved in cancer cell invasiveness (Bajou et al, 1998). These data could explain why MDA-MB-231 cells are more tumorigenic than MCF-7 and MCF-7 ras cells. Surprisingly, an 18 h incubation of cancer cells with NaPa did not modify the expression of u-PA at cell surface and even increased MMP-9 secretion and u-PAR levels, while in the same conditions the invasiveness through Matrigel was dramatically reduced. However when the incubation with NaPa was prolonged for 3 days, a decrease in the expression of u-PA was observed only on MDA-MB 231 cells and only for the highest concentration of NaPa tested (20 mM).

Since an increased cell adhesion could prevent the cell detachment required for their invasiveness, we have investigated cancer cell adhesion on fibronectin and vitronectin, a counterligand of u-PAR, and the expression of LFA-1, ICAM-1 and MAC-1 (CD11b) which are frequently involved in the tumour cell adhesion through the stromal tissue. An 18-h treatment of MDA-MB-231 with NaPa greatly increased cell adhesion to vitronectin. This result can be explained by the increased expression of u-PAR

**Table 3** Effect of neutralizing antibodies against ICAM-1 or LFA-1 on the decreased invasiveness through Matrigel of MDA-MB-231 treated for 18 hours by 20 mM of NaPa

| | Controls | NaPa 20 mM | NaPa 20 mM + anti-ICAM-1 | NaPa 20 mM+ anti LFA-1 |
|---|---|---|---|---|
| % of migrated cells | 100 | 22.2 ± 4.5 | 28 ± 6.3 | 72.6 ± 8.5 |

MDA-MB-231 were treated for 18 hours by 20 of NaPa. After trypsination and 2 washes in PBS, $2\times10^5$ cells were incubated for 30 mn at 4˚C with 100 μg of the neutralizing mAb before to be seeded in the upper chamber of the insert coated with Matrigel. Controls were incubated with an irrelevant IgG1 antibody at the same concentration. After an 18 hour incubation at 37˚C, the cells which had traversed the filter were counted by light microscopy after May-Grünewald coloration. 10 fields were counted for each insert. Results are expressed as the percentage of migrated cells as compared to the controls. (Mean ± SEM of 2 separate experiments.)

© 2001 Cancer Research Campaign on this cell line and this increased adhesivity was suppressed by preincubating the cells with a monoclonal antibody against u-PAR which blocks the interaction with vitronectin (Paysant et al, 1998). This is also supported by the fact that NaPa did not modify the cell adhesion either on vitronectin nor on u-PAR of MCF-7 and MCF-7 ras cells.

Furthermore, the increased expression of LFA-1 is clearly involved in the decreased invasiveness of MDA-MB-231 through Matrigel, since a neutralizing antibody against this integrin partially restores the invasive properties of MDA-MB-231 treated by NaPa. The increase of ICAM-1, even if it is not related to the decreased invasiveness of NaPa-treated MDA-MB-231 could be of importance to explain the antitumoral activity of NaPa, since this molecule can increase host cytotoxic responses against breast tumour (Ogawa et al, 1998). In addition NaPa treatment increased also the expression of MAC-1, another counterligand of ICAM-1, but this increase was delayed as compared to the increase of LFA-1 and ICAM-1. We have previously demonstrated that the cell–cell adhesion of cancer cells was increased by NaPa treatment and that this was explained in part by β cathenin phosphorylation (Thibout et al, 1999). The increase by NaPa of LFA-1, MAC-1 and ICAM-1 could also participate in the increased cell–cell adhesion and the increased MMP-9 secretion since it was shown that cell adhesion molecules regulate secretion of MMP (Edvardsen et al, 1993).

NaPa treatment induced a shape change of MDA-MB-231 cells, which adopted a spindle shape and detached from plastic. This reorganization of the cytoskeleton, as well as the modifications of cell–cell adhesion molecules could be responsible for the increased MMP-9 secretion induced by NaPa since it was recently reported that Paclitaxel treatment of breast cancer cells induces a change in cytoskeleton organization associated with a concomitant increase of MMP-9 secretion (Alonso et al, 1999).

In conclusion, our results suggest that NaPa could affect the complex interactions between ECM proteins and their receptors. By increasing the expression of u-PAR and concomitantly the adhesion to vitronectin, NaPa treatment might also contribute to reduce breast cancer cell invasiveness of aggressive breast cancers which contain high level of the u-PAR/u-PA system (Schmitt et al, 1997). Furthermore, the increase of LFA-1 induced by NaPa contributes also to the decreased invasiveness of NaPa-treated MDA-MB-231. The present results can explain our previous in vivo studies showing that NaPa treatment in nude mice reduces the breast tumour cell invasivity in stromal tissue (Adam et al, 1995). Altogether, these results, with others, strongly suggest that NaPa contributes to the reversibility of a highly invasive breast phenotype by acting on the u-PAR system and on the ECM protein adhesion systems.

### REFERENCES

Adam L, Crepin M, Savin C and Israël L (1995) Sodium phenylacetate induces growth inhibition and Bcl-2 down-regulation and apoptosis in MCF-7 ras cells in vitro and in Nude mice. *Cancer Res* **55**: 5156–5160

Adam L, Crepin M and Israël L (1997) Tumor growth inhibition, apoptosis, and Bcl-2 down-regulation of MCF-7 ras tumors by sodium phenylacetate and tamoxifen combination. *Cancer Res* **57**: 1023–1029

Alonso DF, Farina HG, Arregui C, Aon MA and Gomez DE (1999) Modulation of urokinase-type plasminogen activator and metalloproteinase activities in cultured mouse mammary-carcinoma cell: enhancement by paclitaxel and inhibition by nocodazole. *Int J Cancer* **83**: 242–246

Bajou K, Noel A, Gerard RD, Masson V, Brunner N, Holst-Hansen M, Fusenig NE, Carmeliet P, Collen D and Foidart JM (1998) Absence of host plasminogen activator inhibitor 1 prevents tumor invasion and vascularization. *Nat Med* **4**: 923–928

Bianchi E, Cohen R, Thor A, Todd R, Mizukami I, Lawrence D, Ljung B, Sherman M and Smith H (1994) The urokinase receptor is expressed in invasive breast cancer but not in normal breast tissue. *Cancer Res* **54**: 861–866

Bradford M (1976) A rapid and sensitive method for the quantification of microgram quantities of protein utilizing the principle of protein dye-binding. *Anal Biochem* **72**: 248–250

Carriero MV, Del Vecchio S, Franco P, Potena MI, Chiaradonna F, Botti G, Stoppelli MP and Salvatore M (1997) Vitronectin binding to urokinase receptor in human breast cancer. *Clin Cancer Res* **3**: 12299–12308

Edvardson K, Chen W, Rucklidge G, Walsh FS, Öbrink B and Bock E (1993) Transmembrane neural cell-adhesion molecule (NCAM), but not glycosyl-phosphatidyl-anchored NCAM, down-regulates secretion of matrix metalloproteinases. *Proc Natl Acad Sci USA* **90**: 11463–11467

Hayashi H, Shimizu R, Fujii K, Itoh S, Yang D and Onozaki K (1997) Resistance to IL-1 antiproliferative effect, accompanied by characteristics of advanced melanoma, permits invasion of human melanoma cells in vitro, but not metastasis in Nude mice. *Int J Cancer* **71**: 416–421

Hazan RB, Philipps GR, Qiao RF, Norton L and Aaronson SA (2000) Exogenous expression of N-cadherin in breast cancer cells induces cell migration, invasion and metastasis. *J Cell Biol* **148**: 779–790

Holst-Hansen C, Johannessen B, Hoyer-Hansen G, Romer J, Ellis V and Brünner N (1996) Urokinase-type plasminogen activation in three human breast cancer lines correlates with their in vitro invasiveness. *Clin Exp Metastasis* **14**: 297–307

Loridon-Rosa B, Viehl P, Cuadrado C and Burtin P (1988) Comparative distribution of fibronectin and vitronectin in human breast and colon carcinomas. An immunofluorescence study. *Am J Clin Pathol* **90**: 7–16

Ogawa Y, Hirakawa K, Nakata B, Fujihara T, Sawada T, Kato Y, Yoshikawa K and Sowa M (1998) Expression of intercellular adhesion molecule-1 in invasive breast cancer reflects low growth potential, negative lymph node involvement and good prognosis. *Clin Cancer Res* **4**: 31–36

Ossowski L (1992) Invasion of connective tissue by human carcinoma lines: requirement for urokinase, urokinase receptor, and interstitial collagenase: *Cancer Res* **52**: 6754–6760

Paysant J, Vasse M, Soria J, Lenormand B, Pourtau J, Vannier JP and Soria C (1998) Regulation of the u-PA/u-PAR system expressed on monocytes by the deactivating cytokines IL-4, IL-10 and IL-13: consequences on cell adhesion to vitronectin and fibrinogen. *Br J Haematol* **100**: 45–51

Prasanna P, Shack S, Wilson VL and Samid D (1995) Phenylacetate in chemoprevention: in vitro and in vivo suppression of 5-aza-2′-deoxycytidine-induced carcinogenesis. *Clin Cancer Res* **1**: 865–871

Samid D, Shak S and Sherman LT (1992a) Phenylacetate: a novel nontoxic inducer of tumor cell differenciation. *Cancer Res* **52**: 1988–1992

Samid D, Yeh A and Prasanna P (1992b) Induction of erythroid differentiation and fetal hemoglobin production in human leukemic cells treated with phenylacetate. *Blood* **80**: 1576–1581

Schmitt M, Harbeck N, Thomssen C, Wilhelm O, Magdolen V, Reuning U, Ulm K, Hofler H, Janicke F and Graeff H (1997) Clinical impact of the plasminogen activation system in tumor invasion and metastasis: prognostic relevance and target for the therapy. *Thromb Haemost* **78**: 285–296

Suzuki N, Urano J and Tamanoi F (1998) Farnesyltransferase inhibitors induce cytochrome c release and caspase 3 activation preferentially in transformed cells. *Proc Natl Acad Sci USA* **95**: 15356–15361

Tatsumi T, Shimazaki C, Goto H, Araki S, Sudo Y, Yamagata N, Ashihara E, Inaba T, Fujita N and Nakagawa M (1996) Expression of adhesion molecules on myeloma cells. *J Cancer Res* **87**: 837–842

Thibout D, Di Benedetto M, Kraemer M, Sainte-Catherine O, Derbin C and Crepin M (1998) Sodium phenylacetate modulates the synthesis of autocrine and paracrine growth factors secreted by breast cancer cell lines. *Anticancer Res* **18**: 2657–2662

Thibout D, Kraemer M, Di Benedetto M, Saffar L and Crepin M (1999) Sodium phenylacetate (NaPa) induces modifications of the proliferation, the adhesion and the cell cycle of tumoral epithelial breast cells. *Anticancer Res* **19**: 2121–2126

© 2001 Cancer Research Campaign
